# Characterizing the secret diets of siphonophores (Cnidaria: Hydrozoa) using DNA metabarcoding

**DOI:** 10.1371/journal.pone.0267761

**Published:** 2022-05-20

**Authors:** Alejandro Damian-Serrano, Elizabeth D. Hetherington, C. Anela Choy, Steven H. D. Haddock, Alexandra Lapides, Casey W. Dunn

**Affiliations:** 1 Department of Ecology and Evolutionary Biology, Yale University, New Haven, CT, United States of America; 2 Institute of Ecology and Evolution, University of Oregon, Eugene, OR, United States of America; 3 Integrative Oceanography Division, Scripps Institution of Oceanography, University of California San Diego, La Jolla, CA, United States of America; 4 Monterey Bay Aquarium Research Institute, Midwater Research, Moss Landing, CA, United States of America; University of Connecticut, UNITED STATES

## Abstract

Siphonophores (Cnidaria: Hydrozoa) are abundant and diverse gelatinous predators in open-ocean ecosystems. Due to limited access to the midwater, little is known about the diets of most deep-dwelling gelatinous species, which constrains our understanding of food-web structure and nutrient flow in these vast ecosystems. Visual gut-content methods can rarely identify soft-bodied rapidly-digested prey, while observations from submersibles often overlook small prey items. These methods have been differentially applied to shallow and deep siphonophore taxa, confounding habitat and methodological biases. DNA metabarcoding can be used to assess both shallow and deep species’ diets under a common methodological framework, since it can detect both small and gelatinous prey. We (1) further characterized the diets of open-ocean siphonophores using DNA metabarcoding, (2) compared the prey detected by visual and molecular methods to evaluate their technical biases, and (3) evaluated tentacle-based predictions of diet. To do this, we performed DNA metabarcoding analyses on the gut contents of 39 siphonophore species across depths to describe their diets, using six barcode regions along the 18S gene. Taxonomic identifications were assigned using public databases combined with local zooplankton sequences. We identified 55 unique prey items, including crustaceans, gelatinous animals, and fish across 47 siphonophore specimens in 24 species. We reported 29 novel predator-prey interactions, among them the first insights into the diets of nine siphonophore species, many of which were congruent with the dietary predictions based on tentilla morphology. Our analyses detected both small and gelatinous prey taxa underrepresented by visual methods in species from both shallow and deep habitats, indicating that siphonophores play similar trophic roles across depth habitats. We also reveal hidden links between siphonophores and filter-feeders near the base of the food web. This study expands our understanding of the ecological roles of siphonophores in the open ocean, their trophic roles within the ‘jelly-web’, and the importance of their diversity for nutrient flow and ecosystem functioning. Understanding these inconspicuous yet ubiquitous predator-prey interactions is critical to predict the impacts of climate change, overfishing, and conservation policies on oceanic ecosystems.

## Introduction

The open-ocean is the largest volume of the biosphere habitable by animals [1]. This environment hosts diverse communities and complex food webs [2]. Pelagic food webs sustain manifold fisheries, top predators, and sustain the biological carbon pump [3]. Gelatinous animals play fundamental roles in these food webs [4], acting as herbivores, detritivores, hosts, predators, and prey. The subset of the pelagic food web involving gelatinous fauna has been referred to as the “jelly web” [2]. Among the most abundant [5, 6] and trophically-connected [4] gelatinous predators are siphonophores—mid-trophic organisms that feed on a broad variety of prey such as medusae, salps, crustaceans, molluscs, and fishes [4, 7, 8]. Siphonophores are sit-and-wait, non-visual, ambush predators that rely on prey encountering their tentacles and tentilla [9]. They are abundant and locally diverse colonial cnidarians in open-ocean communities, present in every region of the ocean, with species ranging from above the surface (like the Portuguese man-o-war) to the hadal region (>7000m deep) [10]. In addition, siphonophore aggregations can have significant predatory impacts on larval fish stocks [11].

Progress in elucidating siphonophore diets has been slow due to the intrinsic challenges of working with these animals. Observation and collection of open-ocean taxa requires expensive research vessels and instrumentation to reach their habitat. In addition, siphonophores are extremely fragile, requiring the use of blue water SCUBA divers and Remotely Operated Vehicles (ROVs) to collect them alive and intact [12]. These techniques can be used to collect live specimens for gut content inspection, and video recordings from ROVs allow scientists to observe feeding events. Traditional collection methods such as plankton nets not only break up siphonophore colonies, but can also lead to artifactual ingestions in the cod-end that confound their natural diets.

The diets of some epipelagic siphonophores have been examined through gut content analyses of SCUBA-collected colonies [7, 13], and have been reviewed in Hetherington et al. [8]. Recent studies based on ROV observations have shed some light on the diets of deep midwater siphonophores [4, 8]. However, these approaches are limited by their biases. Visual gut content inspection favors hard-bodied prey that digest slowly, leaving behind diagnostic body parts (i.e. exoskeleton, shell, eyes, etc.). Therefore, soft-bodied, rapidly-digested taxa, such as gelatinous zooplankton, are often underrepresented in dietary assessments. ROVs can observe feeding on gelatinous prey before they become digested. However, ROV observations are skewed towards large prey items that can be easily identified from the camera screen (such as large medusae, ctenophores, crustaceans, or fishes), and can overlook important prey items such as copepods and larvae [8]. In addition, prey are relatively scarce in the open ocean, especially in the deeper regions [2], thus it is infrequent to find specimens capturing prey or carrying visually-identifiable prey in their guts [7].

With the advent of DNA metabarcoding, the diets of many marine predators have been established from gut content DNA [14–17]. These high-throughput amplicon sequencing technologies have extremely high detection sensitivity and bypass the biases posed by visual methods. Recently, the application of DNA metabarcoding to marine predator gut contents has demonstrated the capacity of these methods to detect gelatinous prey [18–22]. In the study of gelatinous zooplankton as consumers, this technology has only been applied to assess the microbial diet of the tunicate *Salpa thompsoni* [23], the predatory diet of the scyphomedusa *Aurelia coerulea* [24], and the diet of the lobate ctenophore *Mnemiopsis leidyi* [25]. However, this technology has not yet been applied to study the diets of siphonophores.

A review of the literature on siphonophore diets revealed significant differences between the diets of epipelagic and deep-dwelling siphonophore species [8]. Gelatinous prey appeared to be more prevalent in deep-sea observations while small crustaceans appeared to be the predominant prey in shallow gut content samples. Since epipelagic species’ diets were exclusively assessed through microscopic gut content inspection and deep-sea species’ diets through ROV observations, it is not possible to determine whether these differences are due to ecological or methodological reasons. To disentangle these confounding factors, it is critical to assess both shallow and deep species’ diets under the same methodological framework. In this case, we believe DNA metabarcoding is an ideal choice, since it can detect both small and gelatinous prey, thus being able to bridge across the methodological shortcomings of visual methods. Here we aim to apply a uniform method to describe diets across the water column as a single, interconnected, pelagic ecosystem.

Siphonophore tentillum and nematocyst morphologies are directly linked to feeding guild. Damian-Serrano et al. [26, 27] used these relationships to generate feeding guild predictions for 45 siphonophore species using their tentillum and nematocyst morphologies as predictors in a discriminant analysis of principal components (DAPC). These feeding guild categories comprise fish specialists (which feed primarily on teleost fish prey), large crustacean specialists (which feed primarily on krill, decapod shrimps, mysids, lophogastrids, amphipods, and other macro-planktonic crustaceans larger than 1cm), small crustacean specialists (which feed primarily on copepods, ostracods, cladocerans, larvae, and other meso-planktonic crustaceans smaller than 1cm), gelatinous specialists (which are able to feed on large gelatinous animals such as salps, ctenophores, or medusae in addition to other zooplankton), and generalists (which feed on a balanced variety of small and large, soft- and hard-bodied prey not including gelatinous animals). These predictions were cast on siphonophore species for which no dietary information was available [27], and thus remained to be tested with new data on siphonophore diets.

Here we use DNA metabarcoding to identify the gut contents of several siphonophore species to obtain more comprehensive insights into their diets. Our primary aims are: (1) Expand the existing knowledge on the diets of open-ocean siphonophores using DNA metabarcoding, (2) qualitatively compare the prey detected by visual and molecular methods to evaluate their technical biases, (3) apply a uniform method to describe siphonophore diets across depth habitats, and (4) evaluate the morphology-based predictions of feeding guilds.

## Materials and methods

### Ethics statement

Our specimen collection and protocol were compliant with all local regulations and under the marine collection permit SC‐191140006, issued to Steven H.D. Haddock by the California Department of Fish and Wildlife. Since no vertebrates or cephalopods were involved, we did not need oversight from an animal care board.

### Siphonophore collection

**Fig 1:**
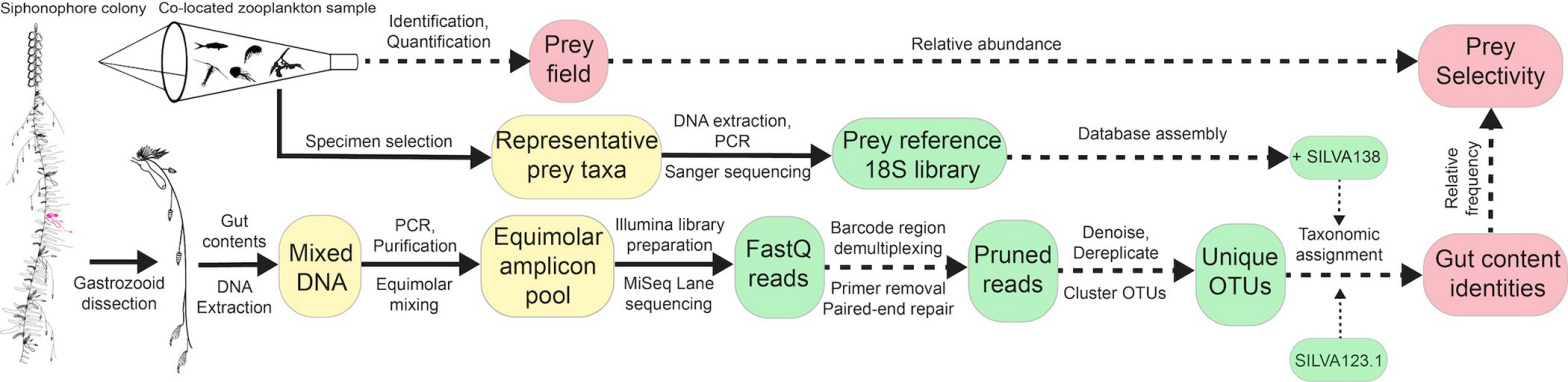
Gut content metabarcoding workflow used in this study. Siphonophore colony illustrated by Freya Goetz. Silhouettes in the plankton net downloaded from phylopic.org. Solid arrows indicate physical material transfer and processing, dashed lines indicate information transfer and processing. Yellow islands indicate elements processed in the laboratory bench, green islands represent bioinformatic datasets processed in the high-performance computing cluster, and red islands represent curated data products.

In order to sample a representative set of taxa across the siphonophore phylogeny, we targeted a set of 41 species (aiming for 10 specimens per species) including cystonects, apolemiids, pyrostephids, euphysonects, and calycophorans from shallow and deep waters. Most species were sampled from the Offshore California Current Ecosystem (OCCE) except for the Portuguese man-o-war *P*. *physalis*, which was collected off Bermuda in the Sargasso Sea; *Sulculeolaria chuni* and some *Nanomia* spp. (labeled as “Atlantic”) which were collected off Rhode Island in the Block Island sound; *Forskalia* sp. M123-SS8 and shallow *Nanomia* sp. KiloMoana2018-BW7-4 which were collected off the coast of Hawaii. While all the *Nanomia* populations sampled in this study have been referred to as *Nanomia bijuga*, we suspect that there may be undescribed cryptic *Nanomia* species among the specimens sampled based on the disparate tentillum morphologies we observed. Therefore, we decided to have them labeled at the genus level. One *Nanomia* specimen (KiloMoana2018−BW7−4) was collected off the coast of Kona, HI. The pleustonic (surface floating) *Physalia physalis* samples were collected manually using a bucket from a small boat. Species found between the 0-20m deep were collected using blue water diving techniques following the guidelines in Haddock & Heine [28]. Species from 200-4000m were collected using ROVs. All animals were collected live and brought back to the ship (or field station in Bermuda for *P*. *Physalis*) for dissection (Fig 1). Live colonies were photographed (sometimes recorded on video), and zooids of diagnostic value (nectophores, bracts, tentacles) were dissected, when possible, fixed in 4% formalin, and stored as vouchers at the Yale Peabody Museum of Natural History (voucher catalog numbers provided in specimen metadata S15 Table).

### Gut content metabarcoding

Shortly after collection of the live specimens, we dissected and pooled several gastrozooids from each colony, making sure that those with visible gut contents are included in addition to several other without conspicuous prey, and also including visible egested food pellets at the bottom of the sampling container. This non-random approach was aimed at increasing our prey detection rate, but may have introduced sampling bias against inconspicuous prey items, thus obscuring any meaningful quantitative analyses of the data. Nonetheless, we still gave inconspicuous prey a broad chance of being represented, since the majority of the gastrozooids we sampled lacked conspicuous prey content. We believe that pooling multiple gastrozooids as a single sample is reasonable, since all gastrozooids in a colony share an actively-flowing, interconnected gastrovascular cavity. Thus, we expected DNA from one prey capture in one gastrozooid to be present within multiple gastrozooids. For small species (such as *Sphaeronectes* spp.), we sampled the whole siphosome (whole colony excluding nectophores) as a pooled collection of all gastrozooids. Samples were frozen at -80°C until DNA extraction.

To extract DNA, we digested the samples with proteinase K at 56°C for 1-2h, and used the DNeasy Blood & Tissue kit (Qiagen, Hilden, Germany) eluting twice at 56°C for 10min into a final volume of 100μl. For barcode amplification, we used a set of six primer pairs that amplify six barcode regions within the 18S gene (‘V3’, ‘V5-V7S’, ‘V5-V7L’, ‘V7’, ‘V7p+V8’, and ‘V9’). The primers were designed using Geneious 11.1.5 [29], constraining the search to short (>300 bp) amplicon products with a high chance of remaining uncleaved after digestion in the gastrozooid, flanked by priming sites conserved (to a maximum mismatch of 3bp) across metazoans. The search for conserved priming sites was conducted on an alignment of 18S genes from 975 species across all metazoan phyla downloaded from GenBank (available in github.com/dunnlab/siphweb_metabarcoding/Primer_design). The primer search was optimized to only retrieve non-degenerate primer pairs with compatible annealing temperatures and without problematic dimerization and hairpin temperatures. Primer sequences are shown in Table 1, and their properties can be found in Table T1 in the protocol (dx.doi.org/10.17504/protocols.io.5qpvo57o7l4o/v2).

**Table 1:** Barcodes used in this study.

Barcode	18S region covered^a^	Forward primer	Reverse primer	Start position^b^	End position^b^
**V3**	Within V3	166F: AACGGCTACCACATCCAAGG	166R: CACCAGACTTGCCCTCCAAT	420	566
**V5-V7S**	Between V5 and the beginning of V7 (short amplicon)	152: TGACGGAAGGGCACCACCAG	152R: TCCACCAACTAAGAACGGCC	1187	1339
**V5-V7L**	Between V5 and the beginning of V7 (long amplicon)	271F: AAACGATGCCGACTAGCGAT	272R: TCCACCAACTAAGAACGGCC	1067	1339
**V7**	Within V7	179F: GGCCGTTCTTAGTTGGTGGA	179R: TGCGGCCCAGAACATCTAAG	1319	1489
**V7p+V8**	Part of V7 and most of V8	261F: AACAGGTCTGTGATGCCCTT	261R: TGTGTACAAAGGGCAGGGAC	1472	1687
**V9**	Within V9	134F: CTTTGTACACACCGCCCGTC	134R: CCTTGTTACGACTTTTACTTCCTCT	1675	1790

^a^The hypervariable region boundaries were annotated following the gene positions defined in Hadziavdic et al. [30].

^b^Start and end positions calculated on the 18S gene sequence of *Lymnaea diaphana* (GenBank accession JF909497.1).

Using these primer pairs, we ran six parallel PCR reactions for each successful extraction, selecting only those which had yielded a DNA concentration above 10ng/l. For each 25μl reaction volume, we used 2 μl of extraction template, 0.5 μl of each primer (at a 10μM concentration), 1μl of BSA, 0.2 μl of GoTaq (Promega, Madison, WI, USA) polymerase and the standard reagents and proportions of the GoTaq kit (Promega, Madison, WI, USA). The thermal cycles included an initial denaturation at 94°C for 2 min, followed by 30 cycles of denaturation at 94°C for 30 s, annealing (variable), and elongation at 72°C for 1 min, followed by final elongation at 72°C for 5 min. For barcode V9, we used an annealing temperature of 48°C for 45s per cycle. For all other barcodes, we used an annealing temperature of 54°C for 60s per cycle. Each batch of reactions for each barcode included a positive and a negative control (the elution buffer used in extraction), and the products were visualized using gel electrophoresis (2% agarose gel dyed with SYBR Safe DNA Stain) to check for amplicon size and monitor the controls. The PCR products were purified using ExcelaPure UF PCR Purification Plates (EdgeBiosystems, Gaithersburg, Maryland, USA). The DNA yield of each purified product was assessed using a Qubit 2.0 fluorometer and the dsDNA High Sensitivity assay (Thermo Fisher Scientific, USA). Purified PCR products from each barcode region for each sample were combined in equimolar pools based on their DNA yield in order to have equal representation in the sequencing lane. Further details on the quality control, PCR mix, and amplicon pooling are fully described in the online protocol (dx.doi.org/10.17504/protocols.io.5qpvo57o7l4o/v2). All molecular bench work was carried out at the Yale DNA Analysis Facility. Amplicon pools were sequenced using Illumina MiSeq (Illumina, San Diego, CA, USA) 250bp paired-end technology (except samples from specimens KiloMoana2018-BW7-4 *Nanomia* sp., D1019-D5 undescribed physonect, D856-SS8 *Stephanomia amphytridis*, D861-D12 *Bargmannia amoena*, D858-D6 *Apolemia lanosa*, and D860-D6 *Erenna sirena* which were sequenced using Illumina MiSeq 150bp) at the Yale Center for Genomic Analysis.

### Prey reference database

In order to enhance the accuracy of the taxonomic assignments of reads, we also built an 18S gene barcoding database of potential prey items to expand on the available reference sequences in public databases. To do this, we collected 60 specimens of 30 species of zooplankton and micronekton from the OCCE using a Tucker trawl. We targeted plausible prey species from motile open-ocean taxa that cohabitate with siphonophores and are underrepresented in SILVA databases, including fishes, crustaceans, jellyfishes, urochordates, chaetognaths, polychaetes, and mollusks. Specimens were photographed alive, then tissue was sampled and frozen, and finally the rest of the animal was fixed in formalin as a voucher to be identified and preserved at the Yale Peabody Museum of Natural History. DNA extraction, quality control, PCR, and amplicon cleanup was carried out in a similar fashion as the metabarcoding protocol described above (and detailed in dx.doi.org/10.17504/protocols.io.5qpvo57o7l4o/v2), using the PCR program with an annealing temperature of 54°C, and a single pair of primers (166F and 134R), spanning the full extent of the sequence containing all barcode regions used in the gut content metabarcoding (from V3 to V9). Purified amplicons were sent in plates with the forward and reverse primer separately for Sanger sequencing from both ends at the Yale DNA Analysis Facility. A total of 89 newly-submitted sequences were then assembled and trimmed at a 95% quality cutoff in Geneious and concatenated with the latest SILVA database (SILVA_138_SSURef_NR99 downloaded on February 23, 2021) pruned to remove non-eukaryotic sequences.

### Bioinformatic pipeline

Amplicon libraries were demultiplexed by primer sequence using custom bash code. Primer sequences were removed using *cutadapt* [31]. The forward and reverse reads were matched and repaired using *bbtools* [32], then denoised and de-replicated using the DADA2 [33] plugin in QIIME2 [34] with a truncation quality threshold of 28. We *de novo* clustered the unique features into operational taxonomic units (OTUs) using the VSEARCH [35] plugin in QIIME2 with a similarity threshold of 95%. To reduce computational load, only the top 100 most abundant features among the clustered OTUs were selected for taxonomic assignment. Taxonomic identities were assigned using the assignment software METAXA2 [36] with a 70% reliability cutoff, comparing the sequences against the SILVA123.1 reference library [37], and against our custom-built library built using SILVA138 as a foundation. The SILVA123.1 database contains 61383 eukaryotic reference sequences, while our custom database (built off SILVA138.1) contains 79044. Animals in the SILVA123.1 taxonomy are annotated to the ranks of superphylum, phylum, subphylum, class, subclass, order, family, genus, and species. However, the SILVA138.1 animal taxonomy was annotated at the levels of clade (e.g. Bilateria, Protostomia, Deuterostomia, Ecdysozoa, Lophotrochozoa), phylum, class, subclass, order, suborder, and species. All bioinformatics analyses were carried out in the Yale High Performance Computing Cluster. The taxonomic assignments and read count data were merged, then parsed to match the sample of origin and the DNA sequence they derived from. Sequence post-processing scripts can be found in the GitHub repository (https://github.com/dunnlab/siphweb_metabarcoding/Scripts).

### Assignment interpretation

Different barcode regions and reference databases displayed different assignment sensitivities for different taxa. Moreover, the two reference databases were annotated at different taxonomic levels, thus revealing unequal assignment reliabilities at different phylogenetic depths. Therefore, the assignment information from different barcodes and reference databases was integrated to interpret the source and taxon of the detected reads. When the assignments from the two databases disagreed or reported suspicious (e.g. non-marine) taxa, we manually checked the sequences in NCBI BLAST. In summary, a combination of annotation database consensus, barcode region consensus, number of reads, manual BLAST checks, and natural history informed priors were used to assign these interpretations.

Taxonomic assignments were manually inspected and annotated with the interpreted consensus taxon and interpreted source (predator, prey, secondary predation, parasite, environmental, unrecognizable sequence, contamination, or cross contamination). Predator sources correspond to the siphonophore DNA from the gastrozooid. These annotations were given to OTUs with typically high read abundances, often taxonomically-assigned as siphonophores, hydrozoans, or unculturable eukaryotes. Prey sources were annotated when plausible prey taxa were assigned. We know that siphonophores can only capture prey that actively swims to trigger tentilla discharge [9, 27]. Therefore, we interpreted that DNA from non-pelagic and/or non-motile organisms cannot be sourced from dietary contributions from microorganisms, marine snow, eggs, or microscopic ciliated larvae. Secondary predation sources correspond to OTUs assigned to animals that were more likely consumed by the co-detected prey than by the siphonophore. For example, crustacean, gastropod, and larvacean sequences in *P*. *physalis* samples were interpreted as secondary predation (prey of their fish prey) given our knowledge on the prey-capture limitations of these animals and the feeding habits of their fish prey.

Parasite interpretations were annotated onto OTUs assigned as trematodes, cestodes, ichthyophonids, and myxozoans, since the most likely explanation for their presence in the samples is due to parasitism in the siphonophores or their prey. We used the environmental category to annotate OTUs likely originated from the microbial community (such as diatoms, dinoflagellates, uncultured eukaryotes) or eDNA (such as rotifers, sharks, ascidians, sponges, bivalves, anemones, echiurids, gastrotrichs, echinoderms, or bryozoans), based on their taxonomic assignment and read abundance. OTUs assigned as ‘uncultured eukaryotes’ were BLAST-checked to differentiate between environmental microbes and failed assignments of siphonophore sequences. We used the contamination category to interpret OTUs assigned to tetrapods (likely from humans), pollen, branchiopods, nematodes, mites, and insects. Amplification experiments on negative controls indicated that these contaminants originated from specimen manipulation in the field and not from the lab bench. The cross-contamination interpretation was used to annotate some suspicious OTUs with low reads in some samples that matched other taxa that were being extracted and amplified at the lab bench. Reads suspected of cross-contamination from other samples amplified in close proximity (assigned to taxa present in the potential sources of contamination, present across multiple samples in the same run with very low read abundances) were conservatively annotated as such.

When the taxonomic assignments from different barcodes disagreed, we annotated the OTUs based on the barcode majority consensus and BLAST checks. For example, Wwen all barcode regions except ‘V5-V7S’ indicate mysid prey but ‘V5-V7S’ identifies a similar number of reads as stomatopod prey, we interpreted those reads as mysid prey. Assignments of shark identities by barcode region ‘V5-V7S’ in one of the *P*. *physalis* samples (specimen BIOS19-D1-P5) were identified as ray-finned fish prey using BLAST searches and interpreted as such, in agreement with the other barcode regions. Assignments of decapod crustacean identities by barcode region ‘V5-V7S’ (in samples D1137-D7 *Forskalia* sp., D1243-BW25 *Diphyes dispar*, and D1244-SS8 *Nanomia* sp.) were interpreted as euphausiid prey in agreement with the assignments on the rest of the barcode regions. The taxonomic composition of the samples was analyzed and visualized in the R programming environment. Scripts and data available in the GitHub repository.

### Prey field characterization

In order to compare the observed diet to the environmental abundances of potential prey taxa, we collected zooplankton and micronekton samples on the same day and station location as the relevant siphonophore gut content samples. The plankton samples paired with epipelagic siphonophore specimens were collected using a weighted hand-held plankton net (ring diameter of 1m for the Bermuda samples, 0.5m for the OCCE and Block Island sound samples) with a mesh size of 250 μm towed for ~10min between 0–20 meters depth at a maximum speed of 1 kt. In order to quantify the deep pelagic community paired with the ROV-collected siphonophore specimens, we collected zooplankton and micronekton samples using a Tucker trawl (frame area: 2 m^2^, mesh size: 500 μm) towed for ~2h between 900 m and the surface at night. Environmental community samples were visually examined live to collect specimens to sequence for the 18S reference library and other purposes, which were annotated as removed. Samples were concentrated using metal sieves and fixed in 4% formalin. Back at the Yale Peabody Museum of Natural History, these samples were visually identified and quantified from a splitter aliquot. Identifications were carried out to the lowest taxonomic level as well as to a broad group level (e.g., copepods, decapods, krill, fish, hydromedusae, chaetognaths, polychaetes etc.). A few individual unaccounted specimens were removed from the haul before preservation to serve other scientific goals during fieldwork, and therefore these samples may be imperfect representations of the community. In order to estimate how selective siphonophore species are for different prey types in the environment, we calculated Strauss Linear Index (LI) [38] at the broad taxonomic group level.


LI=ri−pi (1)

We used this index to capture the difference between the fraction of each prey type in the environment (p_i_) and the observed frequencies of prey types in the gut contents (r_i_).

### Comparisons to published sources

We aimed to compare and expand previous predation results from submersible observations and visual gut content inspections with the new results of DNA metabarcoding of gut contents. Therefore, we used the dietary data compiled in Damian-Serrano et al. [26] from 11 published sources divided into those that used gut content inspections and those that used human- and remotely-operated submersible observations. Many of the submersible observations correspond to ROV observations carried out in the Offshore California Current Ecosystem, spatially overlapping with the location where the majority of our metabarcoding samples were collected. Salps, ctenophores, and medusae were merged into a gelatinous prey type for comparative purposes. Published records for *Apolemia uvaria* were considered equivalent to *Apolemia* sp. for genus level comparisons. Records of all *Forskalia* species were considered equivalent to *Forskalia* sp. To evaluate the morphology-based dietary predictions generated in Damian-Serrano et al. [27], we compared the Bayesian posterior probabilities for each predicted feeding guild for each species to the metabarcoding findings. Small-crustacean guild predictions were mapped to copepod, ostracod, and cladoceran prey. Large-crustacean guild predictions were mapped to decapod, euphausiid, mysid, lophogastrid, stomatopod, and amphipod prey. Generalist guild predictions were mapped to all prey types except gelatinous prey (following the intended distinction with gelatinous specialists used in Damian-Serrano et al. [26]).

## Results and discussion

**Fig 2:**
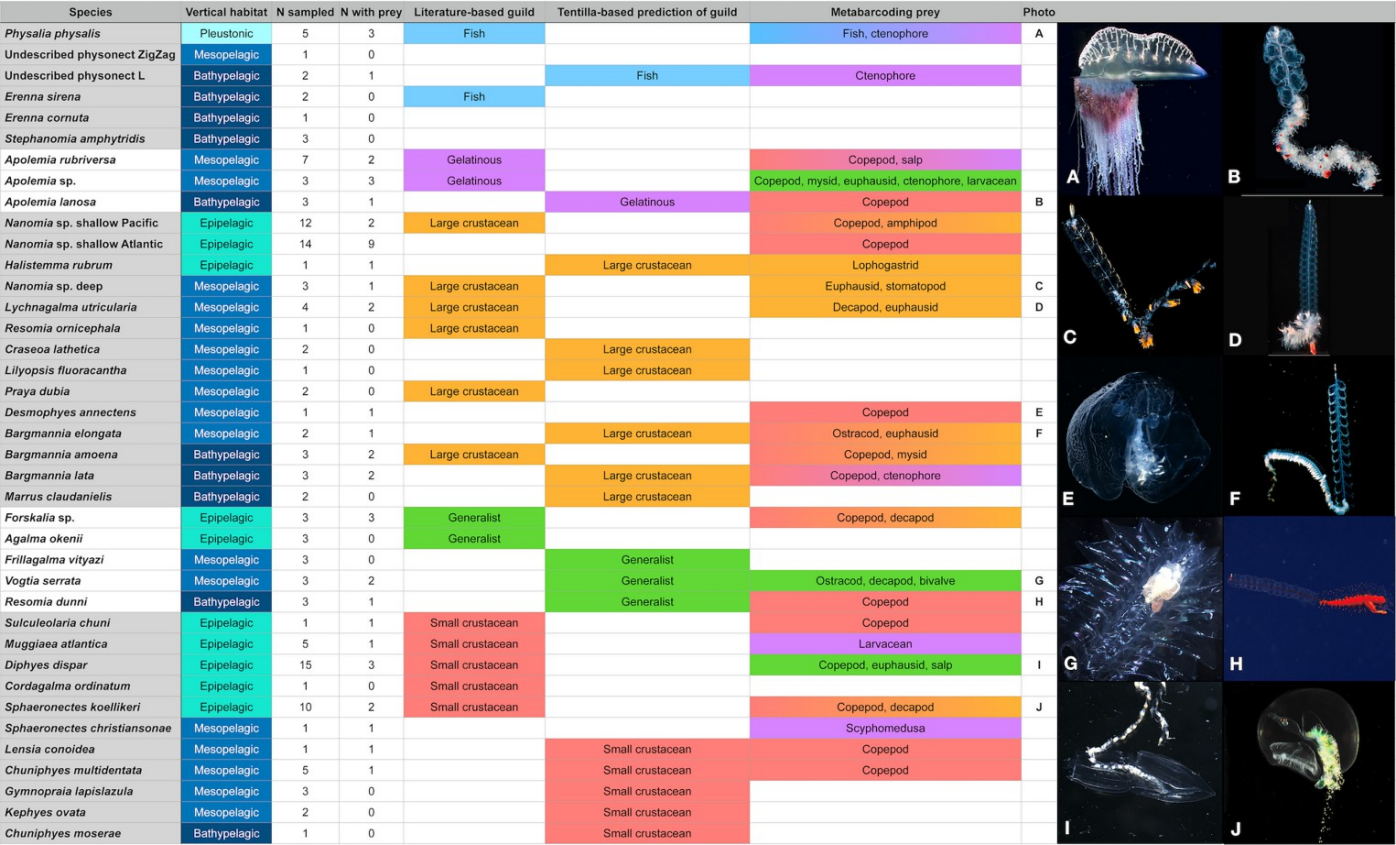
Summary table of the siphonophore species sampled for this study indicating their vertical habitat, the number of specimens sampled, the number of specimens with recognizable prey sequences, and hypothesized feeding guild. Guilds are based on published feeding records used in Damian-Serrano et al. [26], predicted feeding guild from the DAPC analysis in Damian-Serrano et al. [27] based on tentilla morphology, and prey found in this study. Photo credits: (A) Casey Dunn, (B, D,) Stephan Siebert, CC BY licensed and reprinted from Munro et al. [73], (C) reprinted from https://www.theredshrimp.com/ with permission from Reyn Yoshioka, original copyright (2018), (E) Steven Haddock, (F) reprinted from https://biolum.eemb.ucsb.edu/organism/pictures/bargmannia.html with permission from Steven Haddock, original copyright (1997), (G, I) Alejandro Damian-Serrano, (H) NOAA, CC BY licensed, reprinted from https://www.flickr.com/photos/noaaphotolib/19988388271 (J) reprinted from http://www.roboastra.com/Cnidaria2/brac836.htm with permission from Denis Riek, original copyright (2021).

**Fig 3:**
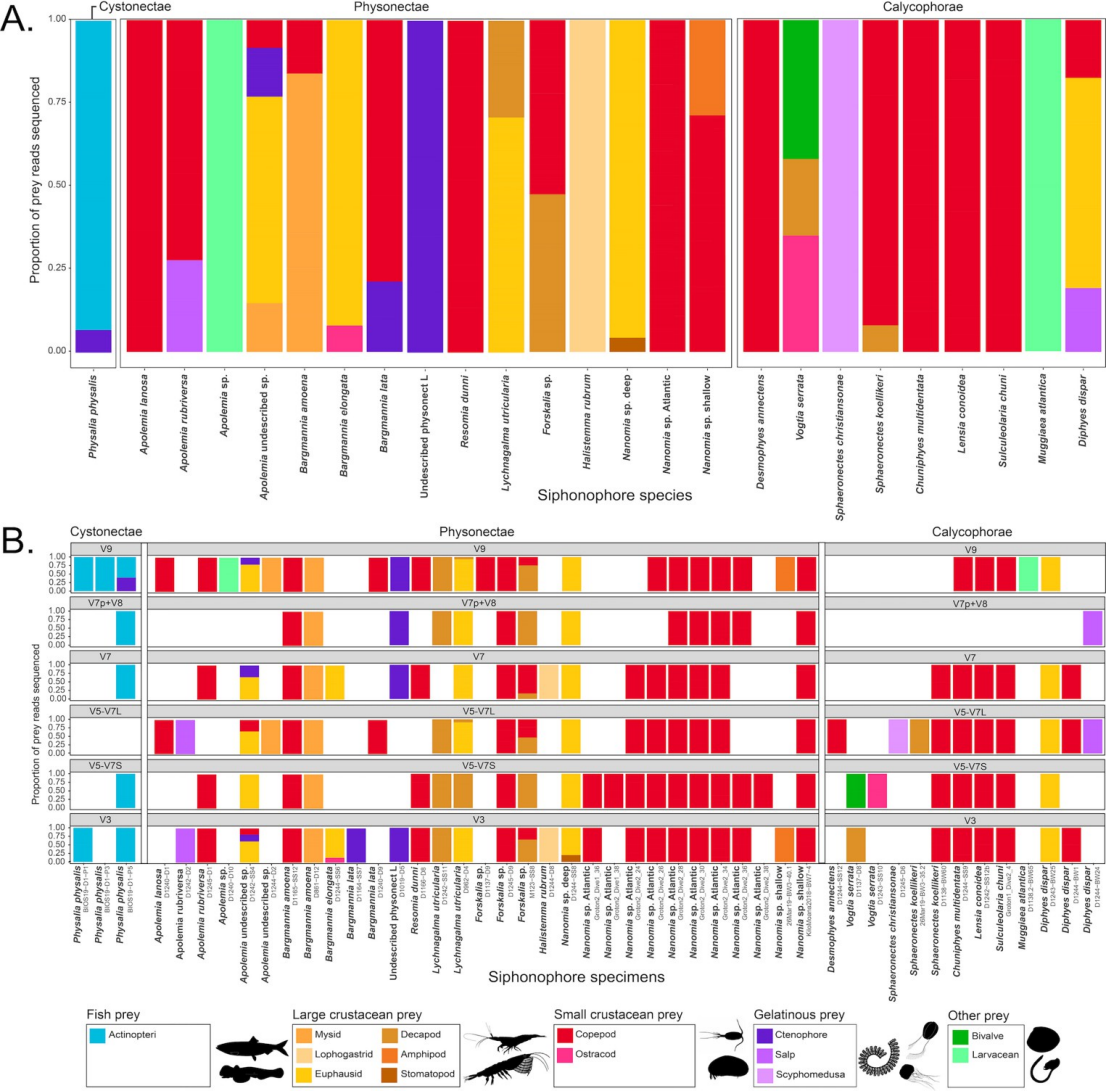
Relative log-abundances of prey reads colored by taxon. (A) For each siphonophore species, and (B) for each siphonophore specimen and barcode.

We extracted, amplified, and sequenced the gut contents of 159 specimens from 41 siphonophore species (Fig 2). We obtained a total of 4148 unique sequences, including 758 from region “V3”, 614 from region “V5-V7S”, 442 from region “V5-V7L”, 497 from region “V7”, and 341 from region “V7p+V8”, and 1502 sequences from region “V9” (S4, S8, S13, and S14 Tables). A total of 337 unique sequences were interpreted as prey items, 36 as secondary predation, 292 as contamination from extrinsic sources, 2857 as natural environmental DNA sources, 791 as siphonophore sequences, 85 as parasites (myxozoans, trematodes, and other helminths), and 14 unrecognizable sequences (S13 and S14 Tables). We identified prey items in 47 specimens (~30%) from 24 siphonophore species (Fig 2, S1 and S3 Tables). This prevalence of empty guts is consistent with the feeding habits of sit-and-wait ambush predators in oligotrophic environments, with scarce feeding events separated by periods of starvation [39]. We identified 55 unique prey items, 42 of which were crustaceans (25 of which were copepods), three of them were fishes, four of them were urochordates, five corresponded to other gelatinous predators (ctenophores and a medusa), and one matching to a bivalve mollusc (Fig 2 and S1 Fig). Most (112 out of 159) siphonophore specimens collected did not yield any putative prey taxa concepts (S3 Table). Among the 47 specimens with prey, 40 of them had DNA from a single prey item, while only six had two prey items, and one *Apolemia* sp. specimen had three prey items (S1 Fig). The use of six different barcode regions with different priming sites and taxonomic specificity allowed us to detect a broader taxonomic range of prey and to validate dubious annotations (Fig 3, S12 Table).

### Dietary findings by taxon

#### Physalia physalis

**Fig 4:**
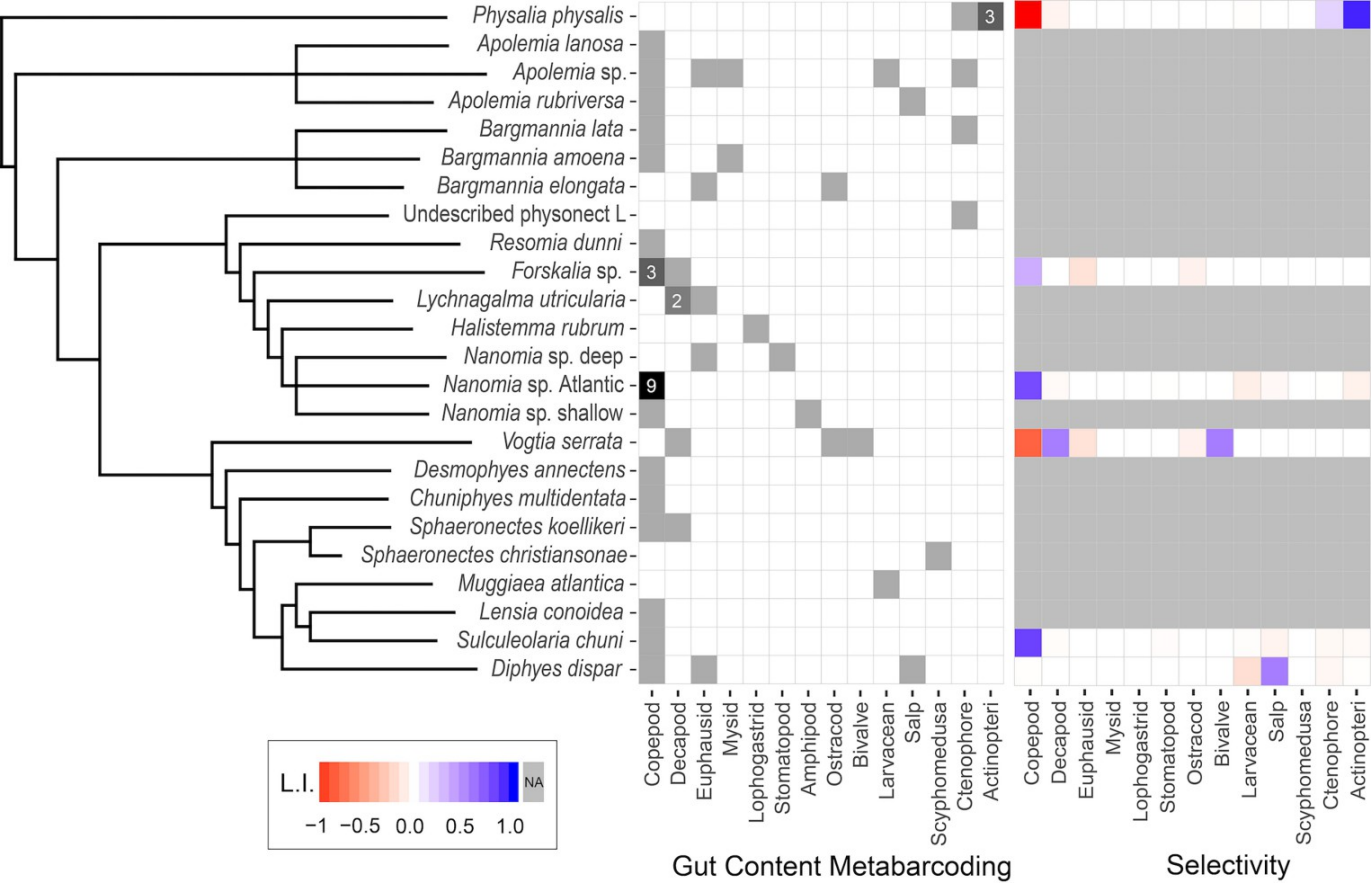
Species-wise grid with the frequency of the major prey types identified from the metabarcoding data and the average prey-type selectivity. Gut content cells in white indicate absence, and cells in grey indicate presence in one specimen, or more than one specimen if labeled with a number. Selectivity colors mapped to Strauss’ L.I. values. The siphonophore cladogram (left) is a simplified version of the phylogenetic tree published in Damian-Serrano et al. [26].

The Portuguese man-o-war is the only pleustonic (surface floating) member of the siphonophores, and the most encountered by beachgoers. Man-o-wars are well-known to feed exclusively on relatively large and motile soft-bodied prey such as fish, chaetognaths, or pelagic gastropods [40]. In our gut content samples of the Portuguese man-o-war from Bermuda, we found three specimens with ray-finned fish sequences (S1 Fig), some of which had visually recognizable fish in the gastrozooids when collected. Fish prey is congruent with published visual inspections of their gut contents [40, 41]. In all three specimens with fish prey we also found benthic and hard-bodied taxa (mysid, alpheid shrimp, spider crab, copepod, benthic gastropod, and a sipunculid worm), as well as larvacean prey sequences (S12 Table). Their nematocysts are not able to subdue crustacean prey, and their feeding reflex would not be triggered by a prey as small as a larvacean or cilia-propelled larva [42]. Therefore, we interpreted the presence of these taxa in the gut contents as secondary predation in the gut contents of the fish prey (S2 and S6 Tables). In addition, we also detected ctenophore prey in one specimen. This could be also a case of secondary predation, but we suspect a ctenophore could be large enough to be prey of the man-o-war. If that is the case, this would be the first record of *P*. *physalis* consuming gelatinous zooplankton, which would place the man-o-war as a central species in the epipelagic ‘jelly-web’ [43]. Comparisons with their surrounding prey field show these specimens were strongly selective for fish and strongly exclusive of copepods (Fig 4).

#### *Apolemia* spp

These are among the longest siphonophores, with colonies attaining lengths as long as 30m [9]. Their tentacles are different from other siphonophores since they have no tentilla and carry birhopaloid nematocysts directly on the tentacles [27]. *Apolemia* species are known to consume diverse prey including crustaceans, molluscs, polychaetes, chaetognaths, fish, and gelatinous zooplankton [4, 7]. While this may suggest these species are generalists, Damian-Serrano et al. [26] hypothesized that they may be gelatinous zooplankton specialists, since they consume a much larger proportion of gelatinous prey than other siphonophores. In addition, the nematocysts of *Apolemia* have similar traits to those in other gelativorous cnidarians [27], and their apparent generality could be explained by the sheer number of fine tentacles deployed for prey capture per colony, which would inevitably entangle almost anything that swims by. We found copepod and salp prey sequences in *Apolemia rubriversa* (Figs 2 and 4). The salp prey found in *A*. *rubriversa* is congruent with its characterization as a gelatinous specialist [26], and may indicate a direct trophic pathway between siphonophores and consumers of microbial production. While the morphology-based predictions indicate that *A*. *lanosa* is likely a gelatinous prey specialist [27], we only found copepod prey in our sample. However, it is possible that the doliolid and hydromedusa reads we conservatively labelled as potential cross-contamination could correspond to real prey. We also analyzed samples from an undescribed *Apolemia* species, where we found a combination of gelatinous (ctenophore), soft-bodied (larvacean), and crustacean (mysid and euphausiid) prey, which is most congruent with a generalist diet (Fig 2). Considering the differences we found between species, it seems possible that these coexisting species of midwater *Apolemia* are partitioning their trophic niche by differentiating the relative proportion of crustacean and gelatinous prey in their diets.

#### *Bargmannia* spp

**Fig 5:**
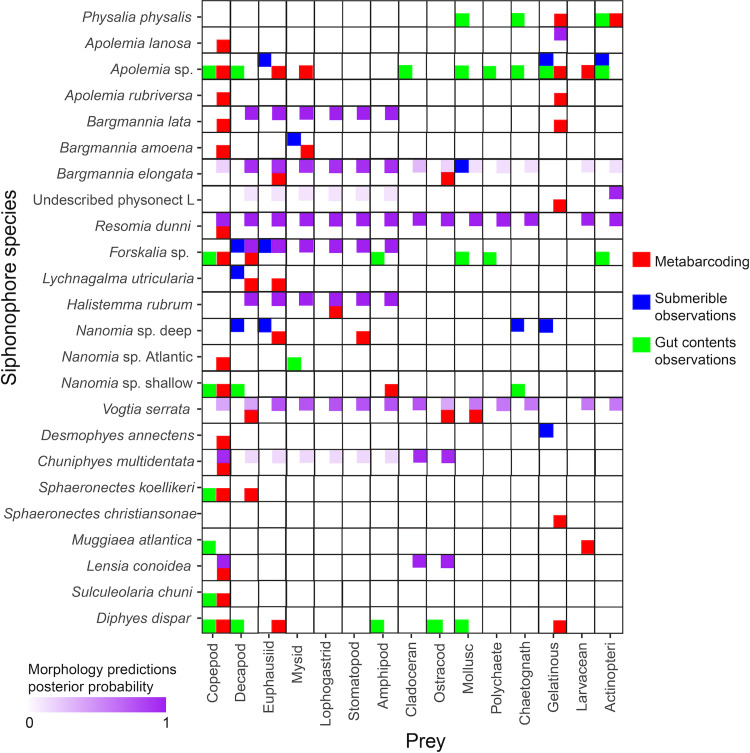
Feeding interactions between siphonophores and their prey from different data sources. Including prey identified by our metabarcoding results (red), observations published submersible observations (blue), observations published visual gut content analyses (green), and prey types predicted by the morphology-based DAPC model in Damian-Serrano et al. [27]. Gelatinous prey refers to ctenophores, medusae, and salps. Larvaceans were excluded as their own category since they are not gelatinous when swimming freely outside their mucous ‘houses’, which would be the only times they would be able to trigger a prey-capture response in siphonophore tentacles.

The three *Bargmannia* species considered here are frequently observed in the midwaters off Monterey Bay, and have relatively simple tentilla with large stenotele nematocysts and an undifferentiated terminal filament [27]. ROVs have recorded *Bargmannia elongata* consuming crustaceans and cephalopods. One *B*. *elongata* specimen had euphausiid and ostracod prey, in agreement with the DAPC prediction Bayesian posteriors for *B*. *elongata* to feed mainly on large crustaceans, but also marginally on small crustaceans (Fig 5). During specimen collection we observed a mysid prey in a specimen *B*. *amoena*. Our DNA metabarcoding identified this mysid as *Boreomysis* (METAXA assignment score 54.68% for V7 on SILVA123.1), and found a copepod in another specimen. Nothing was previously known, however, about the diet of *Bargmannnia lata*. The two *B*. *lata* specimens we sequenced consumed a ctenophore and a copepod, respectively.

The diets of these three closely-related, coexisting species appear to be non-overlapping, which could be a consequence of competitive trophic niche partitioning. The findings for *B*. *lata* consuming copepod and ctenophore prey are not congruent with the morphology-based prediction to be a large-crustacean specialist (Fig 5). We suspect that the lack of taxon sampling among the pyrostephids in [26] could have led to overfitting in the DAPC for this group. Finding ctenophore prey in this species further supports the involvement of deep-sea siphonophores in the midwater ‘jelly web’ hypothesized in Choy et al. [4].

#### Other deep-sea physonects

Undescribed physonect sp. L was predicted to be a fish specialist with a secondary affinity for large crustacean prey (Fig 5). However, we found this specimen consuming a ctenophore. Other morphologically-similar deep-sea undescribed physonects (G and Zigzag) have been observed consuming fish and squid prey [4], thus it is possible that they are specialized in capturing and digesting soft-bodied prey more generally. The rarely-observed *Resomia dunni* was predicted to be a generalist (consumer of all types of prey except gelatinous taxa), which is consistent with the copepod prey we found in its gut contents.

*Forskalia* species are frequently found in both shallow and deep waters, and have been observed to consume various crustaceans, molluscs, worms and fish [7]. However, morphology predicts *Forskalia* species to be large crustacean specialists [27]. We found three midwater *Forskalia* specimens with copepod prey in the guts, one of them also had consumed a sergestid shrimp. These results are fully congruent with those derived from visual methods, and partly congruent with the morphological predictions. *Halistemma rubrum* tentilla closely resemble those of *Forskalia*, and thus they are also predicted to be large-crustacean specialists [27]. This prediction is congruent with our identification of a lophogastrid in the gut contents (Fig 4). On the other hand, *Lychnagalma utricularia* is unique among the physonects for bearing a medusa-shaped floating vesicle at the end of their large, coiled tentilla [27]. They have been observed consuming exclusively large crustaceans through ROVs, such as sergestid shrimp. We found two specimens both with sergestid shrimp prey DNA, yet one of them was also digesting a euphausiid (S1 Fig). This is consistent with previous observations from visual methods and with their hypothesized large-crustacean specialization (Figs 2 and 5).

#### *Nanomia* spp

These are among the most common siphonophores in both Atlantic and Pacific waters, both in epipelagic and midwater environments. We have observed that epipelagic *Nanomia* tend to have smaller tentilla than their mesopelagic counterparts, which may explain their tendency to capture smaller crustaceans such as copepods [7] instead of larger crustaceans such as krill. Midwater ROV observations of deep-dwelling *Nanomia* have predominantly reported interactions with krill prey, as well as with the occasional chaetognath or sergestid shrimp [4]. We identified one specimen of mesopelagic *Nanomia* with krill and stomatopod DNA in its gut contents, congruent with its hypothesized large-crustacean specialist characterization (Fig 5). However, epipelagic *Nanomia* seems to be less specialized on large crustacean prey, since the literature reports a combination of copepod, decapod, mysid, and chaetognath prey [7]. In the North Pacific Ocean, our metabarcoding identified copepod prey in an epipelagic *Nanomia* off California, and a hyperiid amphipod prey in an epipelagic *Nanomia* off Hawaii (S1 Fig). The hyperiid amphipod could have been a commensal or parasite on the *Nanomia* instead of prey, though this is unlikely since only the gastrozooids were dissected while amphipods tend to colonize the nectophores or bracts. In the North Atlantic Ocean, we sampled 14 specimens of epipelagic *Nanomia*, seven of which contained copepod prey (Fig 2). Upon visual inspection of the sampled gastrozooids we could identify *Temora*, *Centropages*, and *Acartia* copepods, the most abundant genera in the plankton sample, whose identity was also supported by the metabarcoding results (genus and species-level assignment scores: *Centropages* sp. 54.99% for barcode V5-V7L, *Acartia tonsa* 91.5% for barcode V5-V7S, and *Temora discaudata* 91.81% for barcode V7p+V8, using the SILVA123.1 database). The corresponding environmental plankton samples showed that these waters were dominated by cladocerans, and thus these *Nanomia* were positively selecting for copepod prey (LI values between 0.69 and 0.72) and selecting against cladoceran prey, which was not detected in the guts (Fig 4 and S1 Fig). The exclusion of the overabundant cladocerans from the diet of Atlantic *Nanomia* suggests that their specialization, if any, could be copepod-specific.

#### Calycophorans

These siphonophores are characterized by their lack of a pneumatophore (gas-filled apical vesicle) and their structurally-homogeneous tentilla [27]. However, these tentilla present a great variation in nematocyst number and size, which may translate into dietary differences [26]. We provided the first insights into the diets of two highly abundant deep-sea calycophorans, *Lensia conoidea* and *Chuniphyes multidentata*, which morphology predicted as small-crustacean specialists. Both sequenced specimens contained copepod DNA (S1 Fig), supporting these predictions (Fig 5). While gelatinous prey has been reported for *Desmophyes annectens* from ROV observations [4], we found only copepod prey sequences. We did find gelatinous prey, however, in *D*. *dispar* (salp prey), and *Sphaeronectes christiansonae* (nausithoid medusa). The latter constitutes the first record of *S*. *christiansonae* feeding. While these medusae can be very small, the minute size of this siphonophore may render this interaction dubious. The far more common epipelagic *Sphaeronectes* species, *Sphaeronectes koellikeri*, appears to be a copepod specialist according to visual gut content analysis, since they appear to feed exclusively on copepods [7]. We sequenced the gut contents of two specimens of this species, one of them indeed was consuming a copepod, yet the other was consuming a crab larva. The latter constitutes a novel prey type for this species, yet still within the expected range of a small-crustacean specialist. Another dietary hypothesis was supported for *S*. *chuni*. Their visually-assessed diet appears comprised exclusively of copepods [7], and we detected copepod prey in a specimen collected in the Atlantic Ocean. On the other hand, while *Muggiaea atlantica* has also been observed feeding exclusively on copepods [7], our specimen had only larvacean sequences that could correspond to prey. While the read abundance for this OTU was low, the small size of this prey type is reasonable given the small tentilla and gastrozooids of *M*. *atlantica* [27, 42].

The calycophoran *Vogtia* is the closest relative to *Hippopodius*, the only siphonophore known to be an ostracod specialist [7]. Like many other hard-to-access mesopelagic taxa, the diet of *Vogtia* has remained unknown, though tentillum morphology predicted them to be generalists [27]. An oceanographic study [44] found spatial correlations between ostracods and *Vogtia* species, and even mentions a *Vogtia* sp. specimen which had the exoskeleton of an ostracod in its gut contents. Our DNA metabarcoding on *Vogtia serrata* has revealed one specimen feeding on an ostracod, and a specimen feeding on a sergestid shrimp and a bivalve (with high selectivity on the latter, LI = 0.5). These results are consistent with the generalist morphological prediction, and congruent with the single visual finding of an ostracod in a congener [44]. The presence of an ostracod and a bivalve (likely a pediveliger larva), which has a very similar shape to an ostracod (with two hard valves), in the gut contents of one of our specimens indicates phylogenetic conservatism of prey traits within Hippopodiidae.

### Comparisons with visual methods

We report the first insights into the diets of nine siphonophore species and reveal 29 novel predator-prey interactions (Figs 2 and 5). When comparing our metabarcoding findings with the published visual observations from gut content inspections and submersible dives, we found five interactions congruent with ROV observations, and eight interactions (six of them involving copepods) congruent with visual gut content inspections of SCUBA-collected colonies (Fig 5).

The published records on the diets of siphonophores appear to differ in prey-type composition between epi- and deep-pelagic habitats [8]. However, the different methodological limitations inherent to each visual method (small prey underestimated by submersibles, soft-bodied prey underestimated by gut content inspections) are hypothesized to be responsible for such differences [8]. Our approach has detected prey types, such as larvaceans, ctenophores, bivalves, and ostracods previously missed by visual methods. The gelatinous animals (i.e. ctenophores, medusae, salps) identified by submersibles as prey of deep-pelagic siphonophores were found present in the gut contents of several deep species (*Apolemia* sp., *B*. *lata*, undescribed physonect L, and *S*. *christiansonae*), supporting the validity of these observations. However, the gelatinous prey recorded by submersibles in prayids such as *Praya dubia* and *D*. *annectens* [4] were not recovered in our *D*. *annectens* samples (Fig 5), suggesting that either our sample sizes were not large enough, or that ROVs had observed accidental entanglement of jellies on their tentacle nets which did not end in ingestion. In addition, we found several small crustaceans in the gut contents of epipelagic species (*Forskalia* sp., *Nanomia* sp., *S*. *koellikeri*, *S*. *chuni*, and *D*. *dispar*) in agreement with visual gut contents observations in shallow habitats. On the other hand, we also found gelatinous and soft-bodied invertebrate prey in shallow-dwelling species (*P*. *physalis*, *D*. *dispar*, and *M*. *atlantica*); as well as small-bodied animals among the prey of deep-pelagic siphonophores (*Apolemia* spp., *Bargmannia* spp., *R*. *dunni*, *V*. *serrata*, *D*. *annectens*, *C*. *multidentata*, and *L*. *conoidea*) (Fig 2). Copepods and ctenophores were the most frequent prey among bathypelagic siphonophores, while other crustaceans (such as ostracods, decapods, and krill) appeared as prey more frequently among the mesopelagic taxa. While these findings are consistent with the hypothesis that small prey is underestimated in submersible observations and rapidly-digested, soft-bodied prey is underestimated by gut content inspections, our sample sizes are insufficient to determine whether the relative contribution of these prey differs between habitats.

DNA metabarcoding was able to detect prey both small and large, gelatinous and hard-bodied, for both deep and shallow-dwelling siphonophore species. These results show that the trophic roles of siphonophores in epi- and deep-pelagic food webs could be more similar than previously-published records may indicate, due to the biases brought by the different diet-assessment methods applied in each habitat. Vertical migration is an important driver of pelagic food web structure [45, 46]. We found copepods, decapods, and euphausiids in the gut contents of both meso- and epipelagic siphonophores. These prey taxa are well-known vertical migrators [47–49], suggesting that there might be some vertical trophic connectivity between these habitats as prey migrates between them. In addition, a few siphonophore species (including *V*. *serrata* and *L*. *conoidea* in this study) are also known diel vertical migrators [50], but their patterns of feeding with depth remain unknown. Finally, our selectivity estimates (for four epipelagic and two mesopelagic species) indicate that siphonophores may play a similar role as selective predators across all depths in the water column.

### Comparisons with prey field

We examined 8 prey-field samples that corresponded to the colocalized ambient prey of 15 out of 47 specimens (some trawls correspond to more than one sampled specimen). The epipelagic plankton samples from Bermuda (colocalized with the *P*. *physalis* specimens) were dominated by copepods, followed by decapod larvae and chaetognaths. While fish larvae were scarce in these samples, they were still far more abundant than in any other sampled location. The Atlantic epipelagic plankton samples (colocalized with the *S*. *chuni* and Atlantic shallow *Nanomia* specimens) were dominated by cladocerans, followed by copepods, larvaceans and salps. The Pacific epipelagic plankton sample from California (colocalized with the *D*. *dispar* specimens) was also dominated by copepods, followed by cladocerans and larvaceans. The quantified midwater tucker trawl from California (colocalized with *V*. *serrata* specimen D1137-D8 and *Forskalia* sp. specimen D1137-D9) was also dominated by copepods (albeit larger species), followed by euphausiids (both adult and larval), chaetognaths, and ostracods.

We found both positive and negative selectivity when comparing identified siphonophore prey to quantified co-localized prey fields. We found strong negative (<-0.5) selectivity for copepods in *P*. *physalis* specimens and in one specimen of *V*. *serrata*. However, in 11 siphonophore specimens from 4 species (out of the 6 species that were quantitatively assessed), we found strong positive selectivity (>0.5) for a specific prey type (S1 Fig). These cases include: selectivity for fish in *P*. *physalis*; selectivity for copepods in *S*. *chuni*, and Atlantic *Nanomia* sp., selectivity for bivalve larvae in *V*. *serrata*, and selectivity for salps in *D*. *dispar* (Fig 4). These selectivity values suggest a strong influence of predator-specific differences in prey capture capabilities for different prey types. However, more replication is necessary in order to test for prey-type specialization.

Epipelagic siphonophores are known to be highly selective and specialized carnivores [7, 26, 51, 52]. ROV observations have revealed that some deep-sea siphonophores are also highly specialized [4]. However, the lack of paired diet and planktonic community samples has limited an assessment of their feeding selectivity. For both the shallow- and deep-dwelling siphonophore species assessed here, we found their prey belonged to the less-abundant components of the co-localized planktonic community, demonstrating high prey-type selectivity. However, the selectivity index values presented in this study should be interpreted with care, since the prey field data is quantitative (abundance-based) but the gut content values are only binary at the specimen level, and frequency-based at the predator species level. Overall, crustaceans (especially copepods) were identified as the most frequent prey type among siphonophore diets. Copepods are typically the most abundant prey type in planktonic communities, thus being able to feed on them is likely an advantageous strategy for any planktivorous predator [53]. Fish prey were detected only in the Portuguese man-o-war samples, in agreement with published observations of man-o-war feeding [40, 41].

Our findings are congruent with the idea that siphonophores span multiple trophic positions, consuming prey across low (salps, larvaceans, copepods, ostracods) and high (fish, ctenophores, medusae) trophic levels. We found larvaceans and salps as prey of shallow- and deep-dwelling siphonophores. These urochordates have an important role in the biological carbon pump, sequestering carbon from phytoplanktonic producers into the deep sea by means of fecal matter production, mucus filter shedding, and carcass depositions [54, 55]. The role of predation on these gelatinous herbivores is often underestimated in oceanic food-web models, or primarily attributed to vertebrate predators [56]. Our results show that some siphonophores like *Apolemia* sp., *A*. *lanosa*, *M*. *atlantica*, and *D*. *dispar* may play an important mid-trophic role incorporating this soft-bodied herbivore productivity into the food web, and providing an alternative avenue to transfer carbon into the deep sea.

### Comparisons with morphology predictions

Comparing our metabarcoding findings with morphology-based predictions [27], we found support for 10 of the 16 predicted interactions between siphonophores and prey. Among the physonects, our results supported the predictions of *B*. *elongata* eating krill and ostracods, *R*. *dunni* eating copepods, *Forskalia* sp. eating decapods, and *H*. *rubrum* eating lophogastrids. Among the calycophorans, we found support for the predictions of *V*. *serrata* eating decapods, ostracods, and molluscs; also *C*. *multidentata* and *L*. *conoidea* eating copepods. Among the siphonophore species studied there were 70 predicted interactions that were not found among the metabarcoding results (Fig 5). Out of the 10 taxa with both morphology-based predictions and metabarcoding results, six had all prey congruent with the predictions, three had all prey incongruent with the predictions, and *Forskalia* sp. presented both cases.

Food-web structure is determined largely by community composition and its patterns in time and space, as the organismal assemblages determine what predators are present and what prey is available to them [57–59]. However, organismal traits constrain which predators can eat which prey [60, 61]. The most commonly-studied trait to predict oceanic food web structure has been size [62, 63]. This is due to the importance of gape size in most predators (i.e. fish, squids, crustaceans etc.) with singular and rigid buccal openings [64, 65]. Siphonophores differ from most predators by having many gastrozooid mouths along their length, all capable of stretching out significantly to ingest prey [66], sometimes utilizing multiple zooids to wrap around large prey [67]. While prey size is still an important constraint for siphonophore-prey interactions [42], siphonophore size is far less relevant. Moreover, some studies have found that phylogenetically-conserved predator traits other than size may also be important predictors of food web structure [68, 69]. Diet is a strong predictor of both extant and ancestral siphonophore tentilla morphology, as well as of its evolutionary dynamics [26]. These relationships were utilized predict the diets of understudied siphonophore species based on the morphology of their tentilla and nematocysts [27]. Here, we were able to test these predictions for ten species and found that most of the prey items found were congruent with these predictions, indicating that tentilla morphology is a strong predictor of siphonophore diets. This finding suggests that some components of the open-ocean food web may be structured by variation in complex morphological traits exclusive to specific predator groups.

Siphonophores are hypothesized to easily evolve between feeding specializations and into a generalist diet due to their modular body plan and their functionally-specialized tentilla [26]. Our results show that closely-related species, such as those within the genera *Bargmannia*, *Apolemia*, and *Nanomia*, appear to feed on different prey. We hypothesize that these species could be further subspecializing to avoid competition or adapt to different prey fields at different depth habitats. This hypothesis is congruent with the conclusions in Damian-Serrano et al. [26] stating that siphonophore dietary evolution can drive rapid morphological shifts. Moreover, we find that *Apolemia* sp., as well as *V*. *serrata*, could be generalists feeding on a variety of crustacean and soft-bodied prey. If a more extensive and quantitative sampling of these taxa was to validate this trophic reclassification, that would suggest that a generalist diet had evolved not just three (as proposed in Damian-Serrano et al. [26]), but up to five times independently, further reinforcing the idea that siphonophore generalists were able to evolve from specialist ancestors.

### Methodological considerations

While DNA-based tools can detect prey unrecognized by visual methods, they are not free of shortcomings. Since all life stages of an animal have the same genetic signature, metabarcoding tools are unable to distinguish between larval, juvenile, or adult prey. These ontogenetic stages can have vastly different ecological implications and pose different challenges during prey capture. In addition, the application of DNA metabarcoding to predator diets is usually not quantitative, since too many sources of variation may lead to differences in read abundance. For example, different animal clades have different sizes, cell densities (due to variable acellular mesoglea content), digestion rates, number of copies of the target gene, or primer affinities during the PCR [70–72]. Due to the difficulties inherent to locating and sampling the species examined in this study, frequency-based quantitative comparisons were not possible for most species either. In addition, the sample size limitations of this study may have biased the results towards higher apparent specialization, and may have missed some important components of the diets of some target species. This caveat is also common in submersible observation data and limits the reliability of comparisons across these methods.

Siphonophores differ from other consumers in several ways which impose further limitations to the value of gut content metabarcoding. The most important aspect is their feeding mode and feeding rate, especially as deep-sea ambush predators, which typically consume one prey at a time and do not get a chance to capture another until far after the former has been digested [9]. Therefore, most siphonophores are found with empty guts or digesting one or few prey items at a time. Thus, the sample size required for frequency-based analyses is much higher than for other consumers which feed more frequently. Moreover, with the exception of a couple species such as *Rhizophysa eysenhardti* and *Rosacea cymbiformis* which are diurnal feeders [7], most species also feed during the night. In the open ocean, diel vertical migration drastically changes the prey field composition for siphonophores at night [45]. Given the fieldwork limitations in this study, we were only able to collect siphonophore gut contents during the day, thus likely biasing their diet towards their diurnal prey captures. Finally, secondary predation (the prey of the prey) cannot be empirically distinguished from direct predation using DNA metabarcoding, and thus we must rely on natural-history based assumptions.

## Conclusions

This study uses DNA metabarcoding technology to investigate the diets of a diverse range of siphonophores. We identified 55 unique prey items in the gut contents of 24 siphonophore species, the majority of which were crustaceans (most of which were copepods), in addition to fishes, molluscs, and gelatinous taxa (Figs 2 and 4). Our results expand the existing knowledge on siphonophore diets, detecting prey types previously missed by visual methods, and providing insights into the diets of several understudied siphonophore species. We show that whole gastrozooids can be utilized for DNA metabarcoding of diets without need for further dissection or the use of predator-blocking primers. We identified representatives from diverse animals (Fig 3, S5–S11 Tables), which demonstrates the phylogenetic range of taxa that can be amplified with our primer pairs. By comparing the taxonomic composition of the gut contents to that of the environmental planktonic community, we find support for the idea that both shallow and deep-dwelling siphonophore species selectively prey on distinct components of zooplankton and micronekton communities (Fig 4). Many of the prey types found in both shallow and deep-dwelling species match published records based on visual methods, but some prey types appear underrepresented by those methods. Moreover, we find that many of the tentillum morphology-based dietary predictions for these species were supported by the metabarcoding results (Fig 5).

Overall, we provide novel insights into the ecology and natural history of several siphonophore species, revealing that siphonophores across all depths are selective predators which have diversified their feeding habits to consume fish, crustaceans, gelatinous predators, gelatinous filter-feeders, meroplanktonic larvae, and other pelagic invertebrates. Our results reveal a significant involvement of deep- and shallow-dwelling siphonophores in the open-ocean ‘jelly web’, highlight suspected biases from visual methods, and support the hypothesized value of tentilla morphology to predict their diets. This study also demonstrates the suitability and effectiveness of DNA metabarcoding to identify the prey consumed by gelatinous predators.

## Supporting information

S1 Fig Species-wise grid with the frequency of the major prey types identified from the metabarcoding data and the average prey-type selectivity. Gut content cells in white indicate absence, and cells in grey indicate presence in one specimen, or more than one specimen if labeled with a number. Selectivity colors mapped to Strauss’ L.I. values.
(TIF)

S1 Table Read abundances assigned to each DNA source interpretation category for each siphonophore species. (TSV)

S2 Table Read abundances assigned to each DNA source interpretation category for each siphonophore species by barcode. (TSV)

S3 Table Read abundances assigned to each DNA source interpretation category for each siphonophore specimen. (TSV)

S4 Table Read abundances assigned to each DNA source interpretation category for each siphonophore specimen by barcode. (TSV)

S5 Table Read abundances assigned to each OTU broad taxon for each siphonophore species. (TSV)

S6 Table Read abundances assigned to each OTU broad taxon for each siphonophore species by barcode. (TSV)

S7 Table Read abundances assigned to each OTU broad taxon for each siphonophore specimen. (TSV)

S8 Table Read abundances assigned to each OTU broad taxon for each siphonophore specimen by barcode. (TSV)

S9 Table Read abundances assigned to each prey OTU broad taxon for each siphonophore species. (TSV)

S10 Table Read abundances assigned to each prey OTU broad taxon for each siphonophore specimen. (TSV)

S11 Table Read abundances assigned to each prey OTU broad taxon for each siphonophore species by barcode. (TSV)

S12 Table Read abundances assigned to each prey OTU broad taxon for each siphonophore specimen by barcode. (TSV)

S13 Table Number of unique sequences assigned to each barcode in each DNA source interpretation category. (TSV)

S14 Table Number of unique sequences assigned to each barcode in each OTU broad taxon. (TSV)

S15 Table Specimen collection metadata and Yale Peabody Museum catalog numbers for voucher specimens. (TSV)

## References

[1] Harbison GR. The gelatinous inhabitants of the ocean interior. Oceanus. 1992;35:18–23.

[2] Robison BH. Deep pelagic biology. Journal of experimental marine biology and ecology. 2004 Mar 31;300(1–2):253–72.

[3] Falkowski PG, Barber RT, Smetacek V. Biogeochemical controls and feedbacks on ocean primary production. Science. 1998 Jul 10;281(5374):200–6. doi: 10.1126/science.281.5374.200 9660741

[4] Choy CA, Haddock SH, Robison BH. Deep pelagic food web structure as revealed by in situ feeding observations. Proceedings of the Royal Society B: Biological Sciences. 2017 Dec 6;284(1868):20172116. doi: 10.1098/rspb.2017.2116 29212727 PMC5740285

[5] O’Brien TD. COPEPOD, a global plankton database: A review of the 2007 database contents and new quality control methodology. 2017 https://www.st.nmfs.noaa.gov/copepod/.

[6] Grossmann MM, Nishikawa J, Lindsay DJ. Diversity and community structure of pelagic cnidarians in the Celebes and Sulu Seas, southeast Asian tropical marginal seas. Deep Sea Research Part I: Oceanographic Research Papers. 2015 Jun 1;100:54–63.

[7] Purcell JE. Dietary composition and diel feeding patterns of epipelagic siphonophores. Marine Biology. 1981 Nov;65(1):83–90.

[8] Hetherington ED, Damian-Serrano A, Haddock SHD, Dunn CW, Choy CA. Moving beyond medusae: Integrating siphonophores into marine food web ecology. Limnology and Oceanography Letters. 2022 Feb Under reviewIn press.

[9] Mackie GO, Pugh PR, Purcell JE. Siphonophore biology. Advances in Marine biology. 1988 Jan 1;24:97–262.

[10] Jamieson AJ, Linley TD. Hydrozoans, scyphozoans, larvaceans and ctenophores observed in situ at hadal depths. Journal of Plankton Research. 2021 Jan;43(1):20–32.

[11] Purcell JE. Feeding ecology of Rhizophysa eysenhardti, a siphonophore predator of fish larvae. Limnology and Oceanography. 1981 May;26(3):424–32.

[12] Haddock SH. A golden age of gelata: past and future research on planktonic ctenophores and cnidarians. Hydrobiologia. 2004 Nov;530(1):549–56.

[13] Biggs DC. Field studies of fishing, feeding, and digestion in siphonophores. Marine & Freshwater Behaviour & Phy. 1977 Jan 1;4(4):261–74.

[14] Leray M, Yang JY, Meyer CP, Mills SC, Agudelo N, Ranwez V, et al. A new versatile primer set targeting a short fragment of the mitochondrial COI region for metabarcoding metazoan diversity: application for characterizing coral reef fish gut contents. Frontiers in zoology. 2013 Dec;10(1):1–4. 23767809 10.1186/1742-9994-10-34 PMC3686579

[15] Harms-Tuohy CA, Schizas NV, Appeldoorn RS. Use of DNA metabarcoding for stomach content analysis in the invasive lionfish Pterois volitans in Puerto Rico. Marine Ecology Progress Series. 2016 Oct 25;558:181–91.

[16] Fernández-Álvarez FÁ, Machordom A, García-Jiménez R, Salinas-Zavala CA, Villanueva R. Predatory flying squids are detritivores during their early planktonic life. Scientific Reports. 2018 Feb 21;8(1):1–2. 29467371 10.1038/s41598-018-21501-y PMC5821876

[17] Van van der Reis AL, Laroche O, Jeffs AG, Lavery SD. Preliminary analysis of New Zealand scampi (Metanephrops challengeri) diet using metabarcoding. PeerJ. 2018 Sep 20;6:e5641. doi: 10.7717/peerj.5641 30258728 PMC6151254

[18] Connell SC, O’Rorke R, Jeffs AG, Lavery SD. DNA identification of the phyllosoma diet of Jasus edwardsii and Scyllarus sp. Z. New Zealand Journal of Marine and Freshwater Research. 2014 Jul 3;48(3):416–29.

[19] McInnes JC, Alderman R, Lea MA, Raymond B, Deagle BE, Phillips RA, et al. High occurrence of jellyfish predation by black‐browed and Campbell albatross identified by DNA metabarcoding. Molecular Ecology. 2017 Sep;26(18):4831–45. doi: 10.1111/mec.14245 28734075

[20] Clarke LJ, Trebilco R, Walters A, Polanowski AM, Deagle BE. DNA-based diet analysis of mesopelagic fish from the southern Kerguelen Axis. Deep Sea Research Part II: Topical Studies in Oceanography. 2020 Apr 1;174.

[21] Jensen MR, Knudsen SW, Munk P, Thomsen PF, Møller PR. Tracing European eel in the diet of mesopelagic fishes from the Sargasso Sea using DNA from fish stomachs. Marine Biology. 2018 Aug;165(8):1–1.

[22] Marques R, Darnaude AM, Crochemore S, Bouvier C, Bonnet D. Molecular approach indicates consumption of jellyfish by commercially important fish species in a coastal Mediterranean lagoon. Marine environmental research. 2019 Dec 1;152:104787. doi: 10.1016/j.marenvres.2019.104787 31522875

[23] Pauli NC, Metfies K, Pakhomov EA, Neuhaus S, Graeve M, Wenta P, et al. Selective feeding in Southern Ocean key grazers—diet composition of krill and salps. Communications biology. 2021 Sep 10;4(1):1–2. 34508174 10.1038/s42003-021-02581-5 PMC8433442

[24] Sun T, Wang L, Zhao J, Dong Z. Application of DNA metabarcoding to characterize the diet of the moon jellyfish Aurelia coerulea polyps and ephyrae. Acta Oceanologica Sinica. 2021 Aug;40(8):160–7.

[25] Schroeder A., Camatti E., Pansera M. and Pallavicini A. Applying DNA metabarcoding for the diet investigation of the invasive ctenophore Mnemiopsis leidyi in a transitional environment. 2022 (Preprint doi: 10.21203/rs.3.rs-1307217/v1).

[26] Damian-Serrano A, Haddock SH, Dunn CW. The evolution of siphonophore tentilla for specialized prey capture in the open ocean. Proceedings of the National Academy of Sciences. 2021a Feb 23;118(8). doi: 10.1073/pnas.2005063118 33593896 PMC7923536

[27] Damian-Serrano A, Haddock SH, Dunn CW. The evolutionary history of siphonophore tentilla: Novelties, convergence, and integration. Integrative Organismal Biology. 2021b May 26. 10.1093/iob/obab019 PMC8331849 34355122

[28] Haddock SH, Heine JN. Scientific blue-water diving La Jolla. CA: California Sea Grant College Program. 2005.

[29] Kearse M, Moir R, Wilson A, Stones-Havas S, Cheung M, Sturrock S, et al. Geneious Basic: an integrated and extendable desktop software platform for the organization and analysis of sequence data. Bioinformatics. 2012 Jun 15;28(12):1647–9. doi: 10.1093/bioinformatics/bts199 22543367 PMC3371832

[30] Hadziavdic K, Lekang K, Lanzen A, Jonassen I, Thompson EM, Troedsson C. Characterization of the 18S rRNA gene for designing universal eukaryote specific primers. PloS one. 2014 Feb 7;9(2):e87624. doi: 10.1371/journal.pone.0087624 24516555 PMC3917833

[31] Martin M. Cutadapt removes adapter sequences from high-throughput sequencing reads. EMBnet. journal. 2011 May 2;17(1):10–2.

[32] Bushnell B, Rood J, Singer E. BBMerge–Accurate paired shotgun read merging via overlap. PLoS one. 2017 Oct 26;12(10):e0185056. doi: 10.1371/journal.pone.0185056 29073143 PMC5657622

[33] Callahan BJ, McMurdie PJ, Rosen MJ, Han AW, Johnson AJ, Holmes SP. DADA2: high-resolution sample inference from Illumina amplicon data. Nature methods. 2016 Jul;13(7):581–3. doi: 10.1038/nmeth.3869 27214047 PMC4927377

[34] Bolyen E, Rideout JR, Dillon MR, Bokulich NA, Abnet CC, Al-Ghalith GA, et al. Reproducible, interactive, scalable and extensible microbiome data science using QIIME 2. Nature biotechnology. 2019 Aug;37(8):852–7. doi: 10.1038/s41587-019-0209-9 31341288 PMC7015180

[35] Rognes T, Flouri T, Nichols B, Quince C, Mahé F. VSEARCH: a versatile open source tool for metagenomics. PeerJ. 2016 Oct 18;4:e2584. doi: 10.7717/peerj.2584 27781170 PMC5075697

[36] Bengtsson‐Palme J, Hartmann M, Eriksson KM, Pal C, Thorell K, Larsson DG, et al. METAXA2: improved identification and taxonomic classification of small and large subunit rRNA in metagenomic data. Molecular ecology resources. 2015 Nov;15(6):1403–14. doi: 10.1111/1755-0998.12399 25732605

[37] Quast C, Pruesse E, Yilmaz P, Gerken J, Schweer T, Yarza P, et al. The SILVA ribosomal RNA gene database project: improved data processing and web-based tools. Nucleic acids research. 2012 Nov 27;41(D1):D590–6. doi: 10.1093/nar/gks1219 23193283 PMC3531112

[38] Strauss RE. Reliability estimates for Ivlevˈs electivity index, the forage ratio, and a proposed linear index of food selection. Transactions of the American Fisheries Society. 1979 Jul;108(4):344–52.

[39] Griffiths D. Prey availability and the food of predators. Ecology. 1975 Aug;56(5):1209–14.

[40] Purcell JE. Predation on fish larvae by Physalia physalis, the Portuguese man of war. Marine ecology progress series. Oldendorf. 1984a Jan 1;19(1):189–91.

[41] Bardi J, Marques AC. Taxonomic redescription of the Portuguese man-of-war, Physalia physalis (Cnidaria, Hydrozoa, Siphonophorae, Cystonectae) from Brazil. Iheringia. Série Zoologia. 2007;97:425–33.

[42] Purcell JE. The functions of nematocysts in prey capture by epipelagic siphonophores (Coelenterata, Hydrozoa). The Biological Bulletin. 1984b Apr;166(2):310–27.

[43] Chi X, Dierking J, Hoving HJ, Lüskow F, Denda A, Christiansen B, et al. Tackling the jelly web: Trophic ecology of gelatinous zooplankton in oceanic food webs of the eastern tropical Atlantic assessed by stable isotope analysis. Limnology and Oceanography. 2021 Feb;66(2):289–305.

[44] Pugh PR. Trophic factors affecting the distribution of siphonophores in the North Atlantic Ocean. UNESCO Technical Papers in Marine Science. 1986;49:230–4.

[45] Sutton TT. Vertical ecology of the pelagic ocean: classical patterns and new perspectives. Journal of fish biology. 2013 Dec;83(6):1508–27. doi: 10.1111/jfb.12263 24298949

[46] Kelly TB, Davison PC, Goericke R, Landry MR, Ohman MD, Stukel MR. The importance of mesozooplankton diel vertical migration for sustaining a mesopelagic food web. Frontiers in Marine Science. 2019 Sep 13;6:508.

[47] Longhurst AR. Vertical Migration. The Ecology of the Seas, Cushing DH and Walsh JJ, eds., Philadelphia: University of Pennsylvania Press; 1976.

[48] Hopkins TL, Flock ME, Gartner JV Jr, Torres JJ. Structure and trophic ecology of a low latitude midwater decapod and mysid assemblage. Marine Ecology Progress Series. 1994 Jun 23:143–56.

[49] Cohen JE, Briand F, Newman CM. Community food webs: data and theory. Springer Science & Business Media; 2012 Dec 6.

[50] Pugh PR. The diel migrations and distributions within a mesopelagic community in the North East Atlantic. 7. Siphonophores. Progress in Oceanography. 1984 Jan 1;13(3–4):461–89.

[51] Purcell JE, Mills CE. The correlation between nematocyst types and diets in pelagic hydrozoa. The biology of nematocysts. 1988:463–85.

[52] Mills CE. Medusae, siphonophores, and ctenophores as planktivorous predators in changing global ecosystems. ICES Journal of Marine Science. 1995 Jun 1;52(3–4):575–81.

[53] Turner JT. The importance of small planktonic copepods and their roles in pelagic marine food webs. Zoological studies. 2004 Jan;43(2):255–66.

[54] Robison BH, Reisenbichler KR, Sherlock RE. Giant larvacean houses: Rapid carbon transport to the deep sea floor. Science. 2005 Jun 10;308(5728):1609–11. doi: 10.1126/science.1109104 15947183

[55] Luo JY, Condon RH, Stock CA, Duarte CM, Lucas CH, Pitt KA, et al. Gelatinous zooplankton‐mediated carbon flows in the global oceans: a data‐driven modeling study. Global Biogeochemical Cycles. 2020 Sep;34(9):e2020GB006704.

[56] Henschke N, Everett JD, Richardson AJ, Suthers IM. Rethinking the role of salps in the ocean. Trends in Ecology & Evolution. 2016 Sep 1;31(9):720–33. doi: 10.1016/j.tree.2016.06.007 27444105

[57] Gotelli NJ, Graves GR. Null models in ecology. 1996.

[58] Ciannelli L, Hjermann DØ, Lehodey P, Ottersen G, Duffy-Anderson JT, Stenseth NC. Climate forcing, food web structure and community dynamics in pelagic marine ecosystems. Aquatic food webs: an ecosystem approach. Oxford University Press, Oxford. 2005 Apr 7:143–69. doi: 10.1098/rspb.2005.3136 16087430 PMC1559850

[59] Cohen JH, Forward RB Jr. Zooplankton diel vertical migration—a review of proximate control. Oceanography and marine biology. 2016 Apr 19:89–122.

[60] Laigle I, Aubin I, Digel C, Brose U, Boulangeat I, Gravel D. Species traits as drivers of food web structure. Oikos. 2018 Feb;127(2):316–26.

[61] Maureaud A, Andersen KH, Zhang L, Lindegren M. Trait‐based food web model reveals the underlying mechanisms of biodiversity–ecosystem functioning relationships. Journal of Animal Ecology. 2020 Jun;89(6):1497–510. doi: 10.1111/1365-2656.13207 32162299

[62] Ward BA, Dutkiewicz S, Jahn O, Follows MJ. A size‐structured food‐web model for the global ocean. Limnology and Oceanography. 2012 Nov;57(6):1877–91.

[63] Zhang L, Hartvig M, Knudsen K, Andersen KH. Size-based predictions of food web patterns. Theoretical ecology. 2014 Feb;7(1):23–33.

[64] Scharf FS, Juanes F, Rountree RA. Predator size-prey size relationships of marine fish predators: interspecific variation and effects of ontogeny and body size on trophic-niche breadth. Marine Ecology Progress Series. 2000 Dec 8;208:229–48.

[65] Costa GC. Predator size, prey size, and dietary niche breadth relationships in marine predators. Ecology. 2009 Jul;90(7):2014–9. doi: 10.1890/08-1150.1 19694148

[66] Pagès F, Madin LP. Siphonophores eat fish larger than their stomachs. Deep Sea Research Part II: Topical Studies in Oceanography. 2010 Dec 1;57(24–26):2248–50.

[67] Hardy SA. The open sea, its natural history, the world of plankton. 1956. No. 574.92 H3.

[68] Gilljam D, Thierry A, Edwards FK, Figueroa D, Ibbotson AT, Jones JI, et al. Seeing double: Size-based and taxonomic views of food web structure. Advances in ecological research. 2011 Jan 1;45:67–133.

[69] Jacob U, Thierry A, Brose U, Arntz WE, Berg S, Brey T, et al. The role of body size in complex food webs: A cold case. Advances in ecological research. 2011 Jan 1;45:181–223.

[70] Deagle BE, Tollit DJ. Quantitative analysis of prey DNA in pinniped faeces: potential to estimate diet composition?. Conservation Genetics. 2007 Jun;8(3):743–7.

[71] Troedsson C, Simonelli P, Nägele V, Nejstgaard JC, Frischer ME. Quantification of copepod gut content by differential length amplification quantitative PCR (dla-qPCR). Marine Biology. 2009 Feb;156(3):253–9. doi: 10.1007/s00227-008-1079-8 32921814 PMC7477863

[72] Valentini A, Miquel C, Nawaz MA, Bellemain EV, Coissac E, Pompanon F, et al. New perspectives in diet analysis based on DNA barcoding and parallel pyrosequencing: the trnL approach. Molecular ecology resources. 2009 Jan;9(1):51–60. doi: 10.1111/j.1755-0998.2008.02352.x 21564566

[73] Munro C, Siebert S, Zapata F, Howison M, Damian-Serrano A, Church SH, et al. Improved phylogenetic resolution within Siphonophora (Cnidaria) with implications for trait evolution. Molecular phylogenetics and evolution. 2018 Oct 1;127:823–33. doi: 10.1016/j.ympev.2018.06.030 29940256 PMC6064665

