# Supplementary figures and images for: Characterizing the secret diets of siphonophores (Cnidaria: Hydrozoa) using DNA metabarcoding
Source: PLoS One. 2022 May 20;17(5):e0267761. doi: 10.1371/journal.pone.0267761 (PMC9122208; doi:10.1371/journal.pone.0267761)

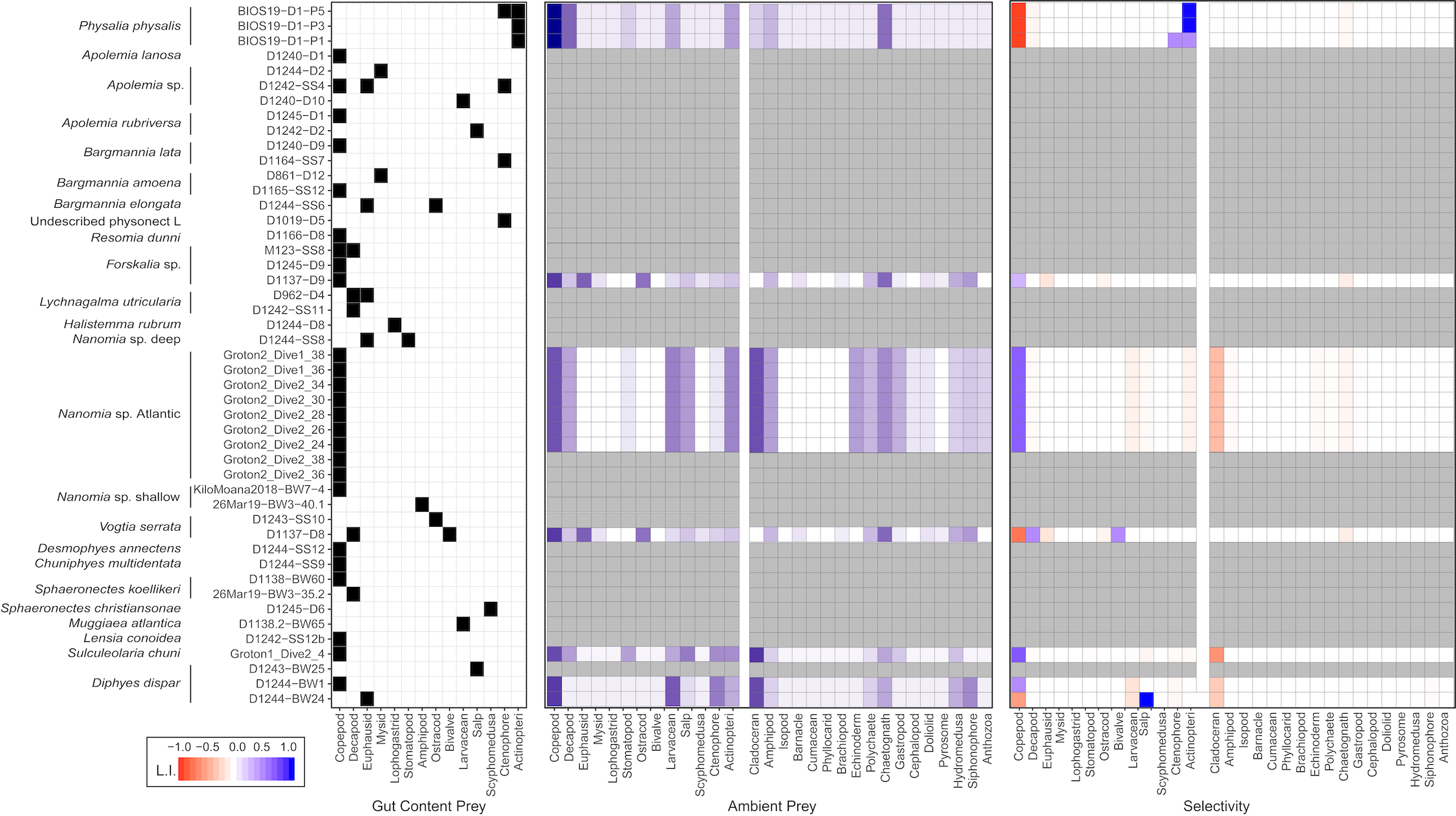

Supplement: S1 Fig — Gut content cells in white indicate absence, and cells in grey indicate presence in one specimen, or more than one specimen if labeled with a number. Selectivity colors mapped to Strauss’ L.I. values. (TIF) [file pone.0267761.s001.tif]
